# The Impact of the Geometric Characteristics on the Hemodynamics in the Stenotic Coronary Artery

**DOI:** 10.1371/journal.pone.0157490

**Published:** 2016-06-16

**Authors:** Changnong Peng, Xiaoqing Wang, Zhanchao Xian, Xin Liu, Wenhua Huang, Pengcheng Xu, Jinyang Wang

**Affiliations:** 1 Department of Cardiology, Shenzhen Sun Yat-Sen cardiovascular hospital, Shenzhen 518112, China; 2 Research center for biomedical information technology, Shenzhen institute of advance technology, Chinese academic of science, Shenzhen 518055, China; 3 Institutes of Clinical Anatomy, Southern Medical University, Guangzhou 510515, China; Magna Graecia University, ITALY

## Abstract

The alterations of the hemodynamics in the coronary arteries, which result from patient-specific geometric significances are complex. The effect of the stenosis on the blood flow alteration had been wildly reported, but the combinational contribution from geometric factors required a comprehensive investigation to provide patient-specific information for diagnosis and assisting in the decision on the further treatment strategies. In the present study, we investigated the correlation between hemodynamic parameters and individual geometric factors in the patient-specific coronary arteries. Computational fluid dynamic simulations were performed on 22 patient-specific 3-dimensional coronary artery models that were reconstructed based on computed tomography angiography images. Our results showed that the increasing severity of the stenosis is associated with the increased maximum wall shear stress at the stenosis region (r = 0.752, P < 0.001). In contrast, the length of the recirculation zone has a moderate association with the curvature of the lesion segment (r = 0.505, P = 0.019) and the length of the lesions (r = 0.527, P = 0.064). Moreover, bifurcation in the coronary arteries is significantly correlated with the occurrence of recirculation, whereas the severity of distal stenosis demonstrated an effect on the alteration of the flow in the upstream bifurcation. These findings could serve as an indication for treatment planning and assist in prognosis evaluation.

## Introduction

Coronary artery disease (CAD) has become the most common cause of death in developing countries. In China, CAD was responsible for 17% of the mortality among the 50 most common causes of death, and this percentage has climbed to above 40% in 2013 [1]. The presence of a coronary arterial lesion is the major contributing factor to adverse clinical outcomes [2]. The anatomic assessment of coronary lesions using medical imaging techniques has been the standard reference for the assessment of ischemic risk. However, the accuracy of the assessment on the basis of medical imaging is relatively debatable such that an over-estimation of stenosis is commonly found when using invasive coronary angiography (ICA) [3,4] and a low specificity for the identification of ischemia-causing coronary stenosis is found when using computed tomography angiography (CTA) [5,6,7]. Although CTA could provide high accuracy in the structural evaluation, functional assessment has been introduced to overcome the discrepancy of myocardial perfusion evaluation [8,4,9]. Fractional flow reserve (FFR) was proposed to meet the requirement of the functional assessment of the vascular bed and to provide physiologic information about the variation in hemodynamics that is associated with coronary arterial stenosis[10]. Considering the amount of non-symptomatic cardiovascular disease and chronic stable angina compared with the occurrence of acute myocardial infarction, it is crucial that early detection and intervention to be implemented in order to prevent further progression to acute clinical symptoms. The functional assessment of the vascular bed using FFR to quantify ischemic risk provides greater accuracy, but it has been applied to less than 10% of all percutaneous coronary intervention procedures, and FFR is utilized to guide management in even fewer cases [3]. The requirement of invasive procedures and the expense of the coronary pressure wire hinder the FFR measurement in clinical implementation [11]. With the advances in computer science, the efficiency and accuracy of numerical simulation have been promoted greatly such that computational fluid dynamics (CFD) analysis in the arterial structures base on the reconstructions from CTA images has been wildly accepted for assisting medical decision-making and prognostic predictions [12]. Taylor et al. developed a novel method for non-invasive FFR measurement based on CTA arterial geometric reconstruction [13]. The accuracy of the calculation compared with the invasive FFR measurement was 86%, but the overall calculation lasted approximately 8 hours. Kwon et al. proposed a simplified procedure in arterial structural reconstruction to achieve an improvement by 42% computational efficiency by reducing the model complexity to a model consisting of coronary arteries and a lumped parameter model boundary condition without the representative component of the aorta [14]. Therefore, based on the reliable anatomic structural analysis using CTA [15,12], the comprehensive evaluation regarding the ischemia of the vascular bed could be achieved for diagnosis.

On the other hand, the progression of the stenosis in the coronary arteries can lead to the increasing the flow velocity at the stenosis and the phenomena of flow reversal at the immediate downstream of the stenosis, ultimately develop into an area of high WSS at the stenosis and a low WSS region downstream, which is also called a recirculation zone [16]. Studies have suggested that a high WSS is correlated with an increase in endothelial permeability, thus allowing the deposition of macromolecules that are susceptible to atherosclerosis [17,18,19]. In contrast, a low WSS value promotes a biological response, which provides an environment for both platelet aggregation and the degradation of the plaque fibrous cap. Thereby increasing the risk of acute clinical symptoms through thrombosis and rupture of the plaque [20,21].

The purpose of the present study is to investigate the effect of the patient-specific geometries combine with individual stenosis on the variation of the hemodynamics in the patient-specific coronary arteries. Correlation analysis has been performed between the geometric parameters and the hemodynamic parameters. Distributions of the flow in the coronary arteries in response to the specific geometries are also investigated to provide a comprehensive understanding on the hemodynamics and further information for the diagnosis.

## Material and Methods

### Populations

The present study was approved by the ethical review committee of the Shenzhen Sun Yat-Sen cardiovascular hospital (Shenzhen, Guangdong, China). The written informed consent forms were obtained from all of the patients. The patients were excluded if any of the following criteria was met: total occlusion of the coronary arteries; previous myocardial infarction; unable to provide inform consent; presented acute symptoms in the previous 60 days; unable to receive adenosine or iodine-based contrast media; had arrhythmia; had calcifications; or had previously undergone coronary artery bypass graft surgery or percutaneous coronary intervention. Therefore, 19 patients in total were included in the present study. The average age ranged from 50 to 74 years old (65 years old ± 17 years), and the patients were diagnosed with the cardiovascular disease between March 5, 2015, and July 7, 2015.

### Image acquisition and FFR measurement

The data was collected following the same standard data acquisition protocol. CTA had been performed in each patient, and the functional assessments were performed at the lesion sites using invasive FFR. In particular, coronary CTA was performed following the Society of Cardiovascular Computed Tomography guidelines [22]. CTA data at both systole and diastole were recorded. The severity of coronary lesions with a degree of stenosis in the main coronary arteries above 50% was considered significant, and ICA was performed according to a standard protocol [16]. Validation of our CFD results was provided by evaluating the relationship between the coronary physiology measurements and calculations. The gold standard in the functional assessment of the coronary blood perfusion is the fraction flow reserve (FFR). A pressure-temperature sensor guidewire was used to obtain physiology measurements in significant stenosis. The pressure measurement starts from a position at least 3cm downstream of the lesion. Hyperemia was induced using an infusion of adenosine (140 μg·kg^-1^·min^-1^) via the femoral vein [1]. The proximal arterial pressure (Pa) and distal arterial pressure (Pd) were recorded, and FFR was calculated by dividing the mean distal coronary pressure (mPd) by the mean aortic pressure (mPa) during hyperemia. The FFR data along the pressure wire retraction and diagnostic FFR value were recorded as the gold standard for the assessment of the CTA-based FFR estimations.

### Model establishment

Patient-specific coronary arterial geometries were reconstructed from 19 sets of CTA image data. 22 coronary arteries (left coronary artery and right coronary artery) were reconstructed from the CCTA data, and 22 lesions were identified with stenosis by anatomic evaluation, derived by dividing the cross-sectional area of the stenosis to the cross-section area at the healthy segment of the proximal to the stenosis. The severities of the lesions ranged from 11% to 90% (mean ± standard deviation is 45% ± 26%) such that 9 out of the 22 lesions were considered to demonstrate greater than 50% stenosis. A previous CFD study of patient-specific coronary arterial geometries showed no significant differences in analyzing the blood flow distributions between the reconstructed model with and without the component of the aorta [14]. To achieve the efficacy, patient-specific arterial geometries in the present study were established with coronary arterial components only. Details of the coronary geometries were determined by the distribution of contrast agent, and the data of diastole were used for geometric reconstruction because the coronary lumen was compressed during systole such that the geometry of the vessel was not distinguishable to the surrounded tissue. Vessels were reconstructed offline using the 3-D reconstruction software Mimics (Materialise NV, Leuven, Belgium). The coronary arterial branches were semi-automatically tracked by the cardiac & vascular module. The geometric models were generated for CFD analysis using nonstructural meshing with tetrahedron elements. Different densities of the meshes were generated in one model for the convergent test such that the CFD results were independent of the finite element discretization that the density ranged from coarse (approximately 17,100 nodes with 85,600 elements) to fine (approximately 32,800 nodes with 545,820 elements). The maximum velocities from the calculation were considered indexes such that convergence was obtained when the difference between values from the models of two mesh densities was lower than 0.1%. The volumes of the geometries varied individually, the mean ± standard deviation of the element numbers of all geometries was 598,371 ± 103,490.

### CFD configuration

The present study focus on the hemodynamics in the coronary artery at the peak flow velocity phase, the flow distribution was assumed full-developed. Therefore, flow for the simulation was assumed to be incompressible, laminar and Newtonian [23]. The walls of the vessels were assumed to be rigid with no-slip boundaries. Incompressible Newtonian fluid assumed that the blood viscosity and density were constant at 0.0035 Pa∙s and 1056 kg/m^3^, respectively. The discreted coronary flow rate was implemented in the inflow boundary. The lumped parameter model (LPM) from the previous study, consisting of resistors and compliance, was implemented in the outflow boundaries [14], and the parameters of LPM were calculated following the same procedure. The vascular resistance values were derived from the total coronary flow rate and cross-sectional area of the branches and from the compliance values [24]. The flow momentum and mass conservation were solved using the following Navier-Stokes governing equations:

$$\rho \left(\frac{du}{dt}+u∙\nabla u\right)=-\nabla p+\mu \nabla^{2}u+f$$ (1)

−∇∙u=0, (2)

where ρ is the density of blood, u is the velocity field, p is the pressure, μ is the viscosity. All data were collected while the patients were at rest, the external force was not involved so that the body force per unit volume f was taken to be zero [25]. Simulations were performed using COMSOL Multiphysics (COMSOL AB, Stockholm, Sweden). The reconstructed geometries of the coronary arteries were implemented and a multifrontal massively parallel sparse direct solver (MUMPS) was used for the simulations [26].

### Quantification of the parameters

**Fig 1:**
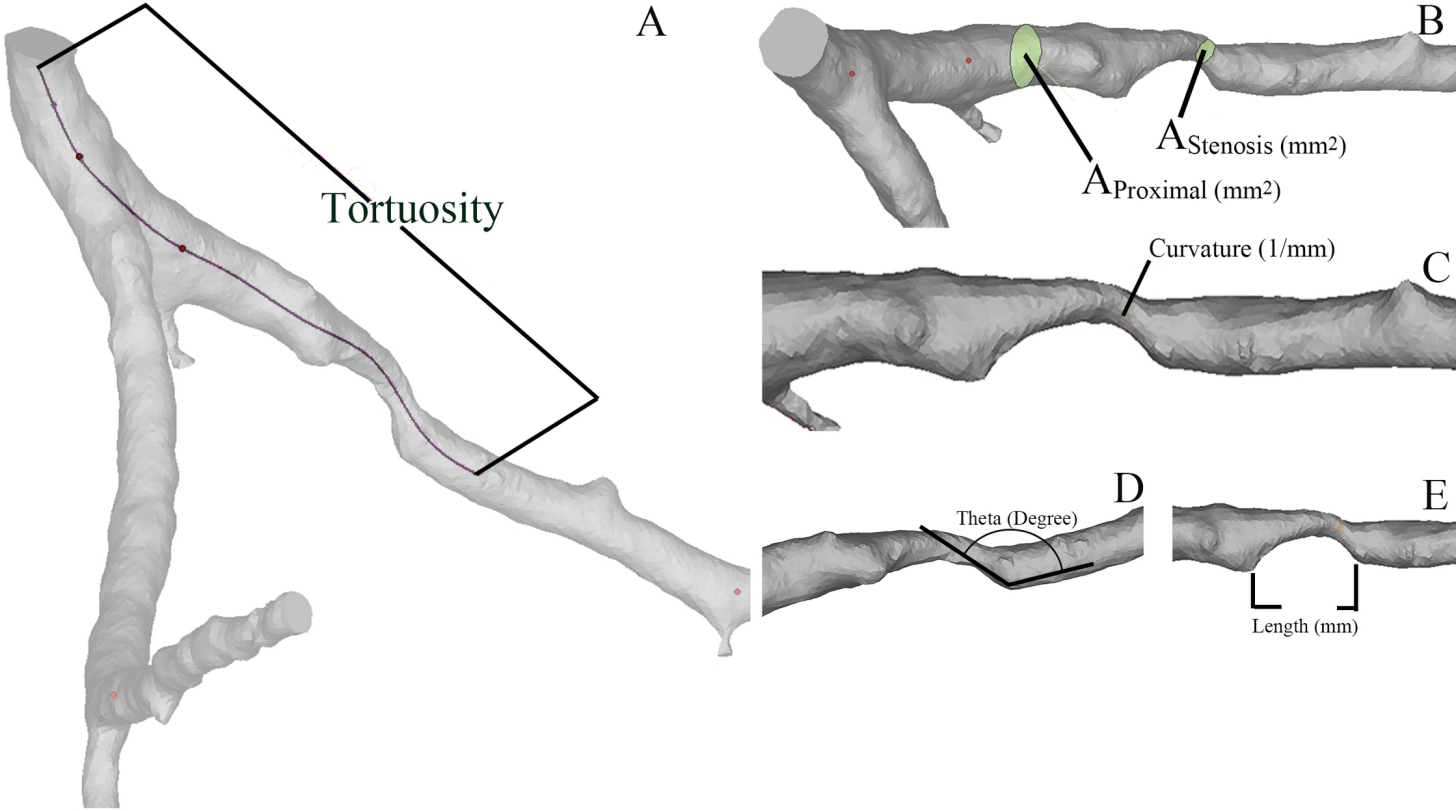
The geometric characteristics include severity of stenosis, tortuosity of the lesion segment, curvature of the lesion segment, angle of the lesion and length of the lesion. A: Tortuosity (T) was taken as the ratio of the length from the ostium of the coronary segment to 1 cm distal to the stenosis (L) to the distance between the ends of the segment (C). B: The severity of the stenosis is the ratio of the cross-section area in the remain lumen at the stenosis and the healthy proximal segment. C: Curvature (unit: 1/m) of the stenotic artery segment was measured, of which the ends of the segment were 1cm proximal to the stenosis and 1cm distal to the stenosis. D: The angle of the lesion was measured at the immediate distal edge of the lesion (unit: degree). E: The length of the lesion was also measured (unit: mm).

Geometric parameters were calculated from the reconstructed models for geometric analysis (as showed in Fig 1). These geometric characteristics include severity of stenosis, tortuosity of the lesion segment, the curvature of the lesion segment, the angle of the immediate downstream of the lesion and length of the lesion. The severity of the stenosis is the ratio of the cross-section area of the remaining lumen at the stenosis (A_Stenosis_) and the healthy proximal segment (A_Proximal_), (Severity = A_Stenosis_ / A_Proximal_). Tortuosity (T) was taken as the ratio of the length from the ostium of the coronary segment to 1cm distal to the stenosis (L) to the distance between the ends of the segment (C), (T = L / C). Curvature (unit: 1/m) of the stenotic artery segment was measured at the peak stenosis. The angle of the immediate downstream of the lesion was measured at the immediate distal edge of the lesion (unit: degree). The length of the lesion was also measured (unit: mm).

The impact of the stenosis on the blood flow distributions in the coronary arteries was also investigated. Hemodynamic parameters were collected from the simulations. These parameters including FFRCT, wall shear stress(WSS), and the length of the recirculation zone. The FFRCT was the ratio of the pressure value at the stenosis to the pressure value at the ostium of the coronary artery. The wall shear stress was calculated from the product of the shear rate and viscosity. The two ends of the recirculation zone were marked in the results, then the length of the recirculation was calculated as the absolute distance between the ends of the recirculation zone.

### Statistical analysis

The results are expressed as the means ± SD unless otherwise stated. Spearman's correlation was performed to investigate the relationships between continuous parameters that did not follow a normal distribution. Bland-Altman plots were used to compare FFR measured by thermodilution with CFD-derived FFRCT. Statistical analyses were performed using SPSS (version 15, SPSS, Chicago, IL). Two-sided P values less than 0.01 were considered statistically significant.

## Results

### Validation of CFD

**Fig 2:**
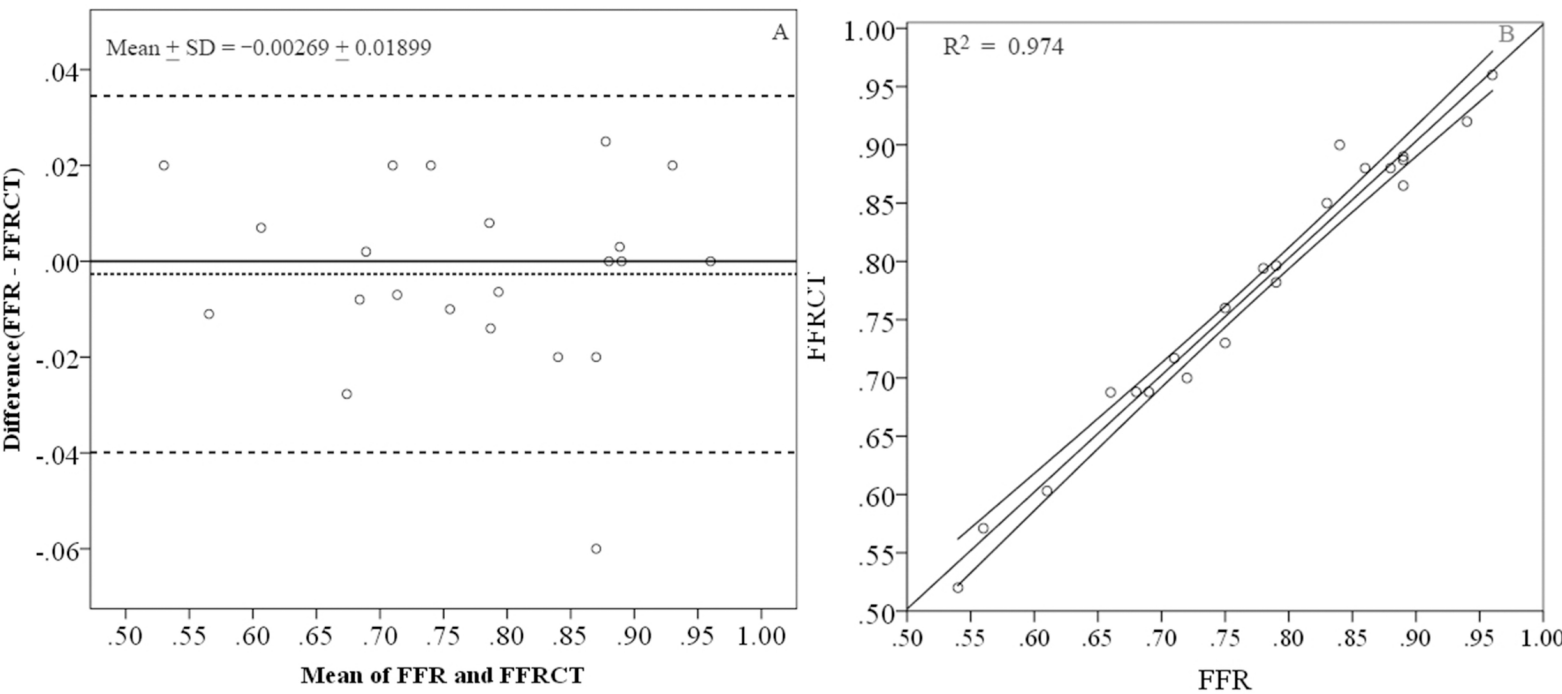
Validation of the calculation is provided by comparing calculated FFRCT to the measurement FFR. A: Agreement between the FFRCTA and FFR is evaluated by Bland-Altman agreement test, the mean±SD bias of is 0.00269 ± 0.01899. B: Agreement between FFRCT and FFR is also evaluated by linear regression that R^2^ = 0.974 with 95% confident interval.

The calculation was validated by comparing CFD-derived FFRCT to the measured FFR. As illustrated in Fig 2, the Bland-Altman agreement test shows the limits of agreement for FFR values, with a mean ± SD bias of -0.00269 ± 0.01899 (Fig 2A). Good agreement was observed between calculations and the measurements (R^2^ = 0.974 with 95% confident interval as showed in Fig 2B), and significant correlation was found that the Spearman correlation factor equaled to 0.987 (P<0.01).

### Correlation of the geometric characteristics to the pressure distribution, the WSS, and the recirculation zone

We investigated the effect of the geometric characteristics on the pressure distribution in the coronary arteries, the maximum WSS at the stenosis and the length of the recirculation zone. These geometric characteristics included the severity of stenosis, tortuosity of the lesion segment, the curvature of the lesion segment, the angle of the immediate downstream of the lesion and length of the lesion.

**Fig 3:**
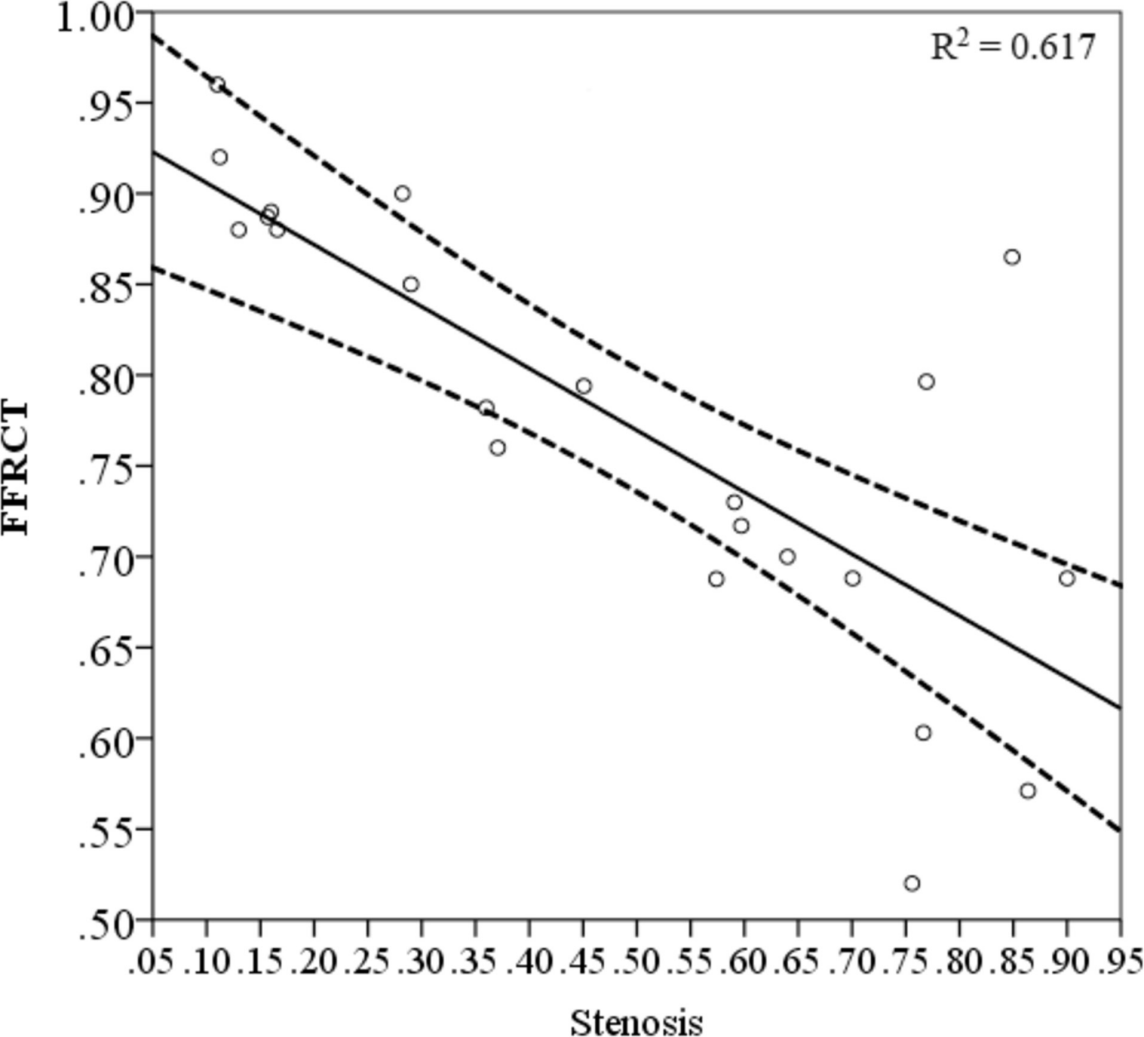
The correlation analysis of FFRCT and the severity of the stenosis. The correlation factor R2 was 0.617 with 95% confident interval. 19 out 22 cases were included in the confident interval and close to the range while 3 cases showed diversion.

**Fig 4:**
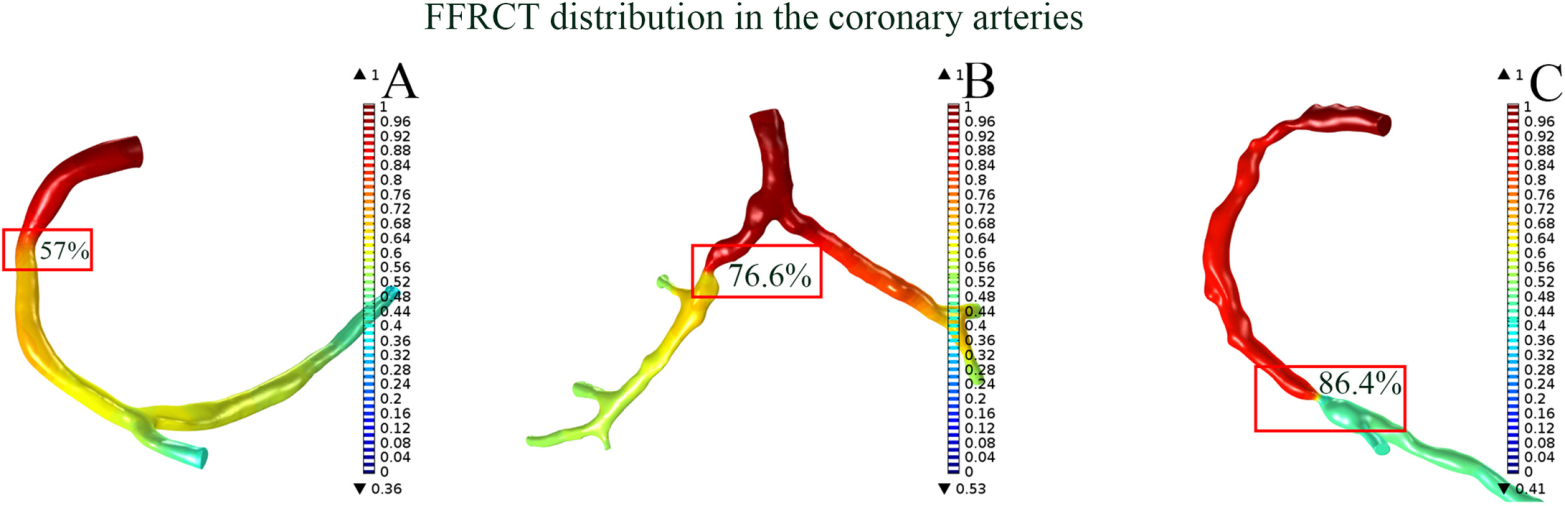
The FFRCT distribution in the coronary arterial geometries with stenosis. The FFRCT at the stenosis decreased as the severity of the stenosis increased. Degree of the stenosis in A, B and C was 57%, 76,6% and 86.4%, the corresponding FFRCT value was 0.687, 0.603 and 0.57, respectively.

**Fig 5:**
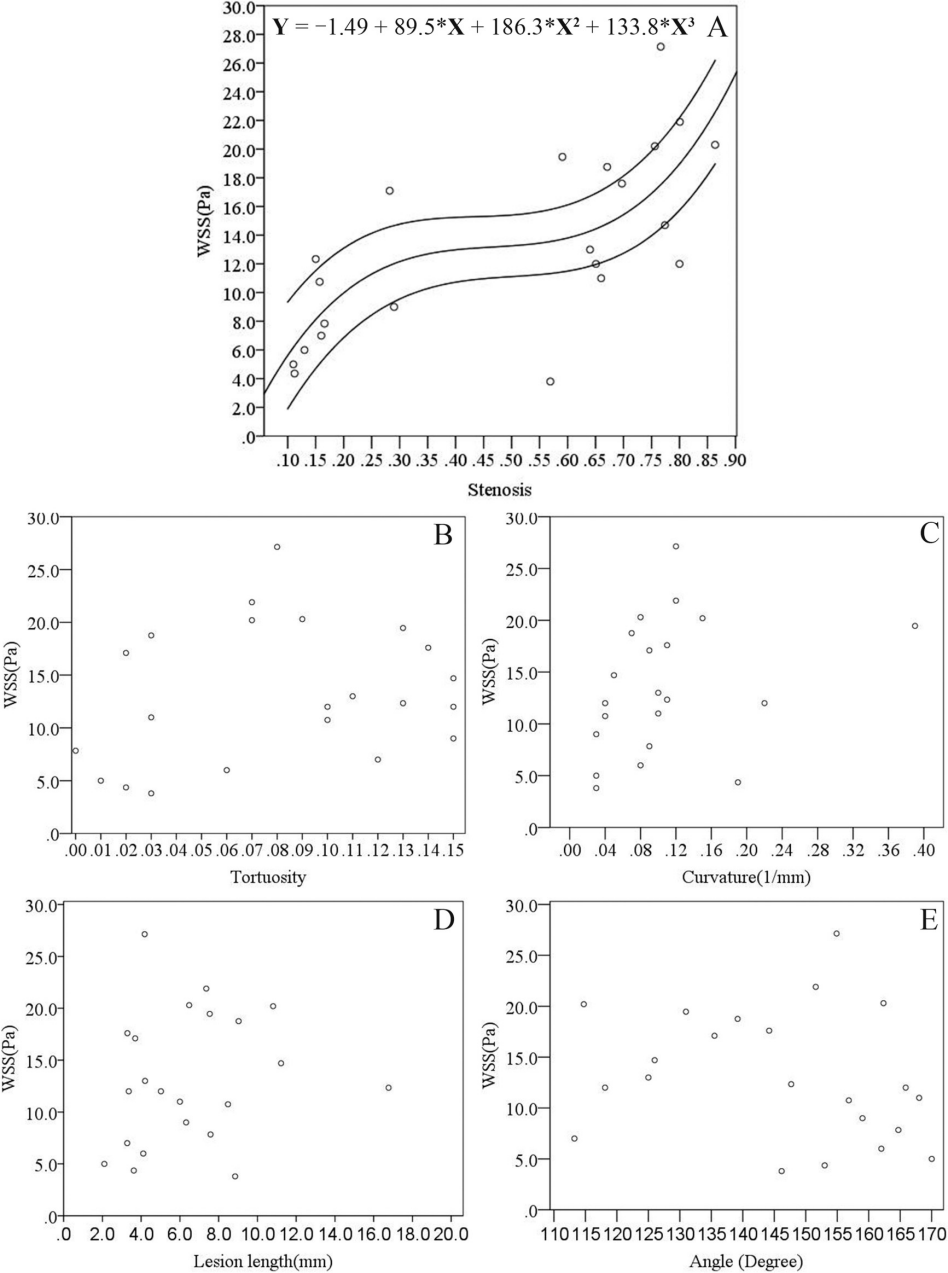
Relationship among stenosis severity, tortuosity, curvature, angle, lesion length with wall shear stress in the reconstructed patient-specific coronary arteries. A: effect of stenosis severity (percent diameter stenosis) on maximum wall shear stress; B: effect of tortuosity on maximum wall shear stress; C: effect of curvature on maximum wall shear stress; D: effect of lesion length on maximum wall shear stress; E: effect of angle of the lesion segment on maximum wall shear stress. Third-order nonlinear cur fit with 95% confident interval is shown in A with R^2^ equals 0.521.

The Spearman correlation between FFRCT and the severity of stenosis was found significant (r = -0.786, P < 0.01, R^2^ = 0.617 with 95% confident interval as showed in Fig 3). The FFRCT at the stenosis decreased as the severity of the stenosis increased (as showed in Fig 4). However, the FFRCT was found was no significant correlation to the tortuosity (r = -0.366, P = 0.094), curvature (r = -0.276, P = 0.214), angle (r = 0.188, P = 0.402), and the length (r = -0.384, P = 0.078). The tortuosity (r = 0.264, P = 0.234), angle (r = -0.286, P = 0.197) and length (r = 0.268, P = 0.227) had no significant impact on the maximum WSS at the lesion, as shown in Fig 5B, 5D and 5E respectively. In contrast, the severity of the stenosis contributed to the increase in the maximum WSS at the lesion, as showed in Fig 5A (r = 0.766, P < 0.01). There was a trend of a steep increase in the WSS at both mild stenosis (0% to 30%) and severe stenosis (60% to 90%). Further, the curvature of the lesion segment was correlated to the increase in the maximum WSS, as showed in Fig 5C (r = 0.457, P < 0.05).

**Fig 6:**
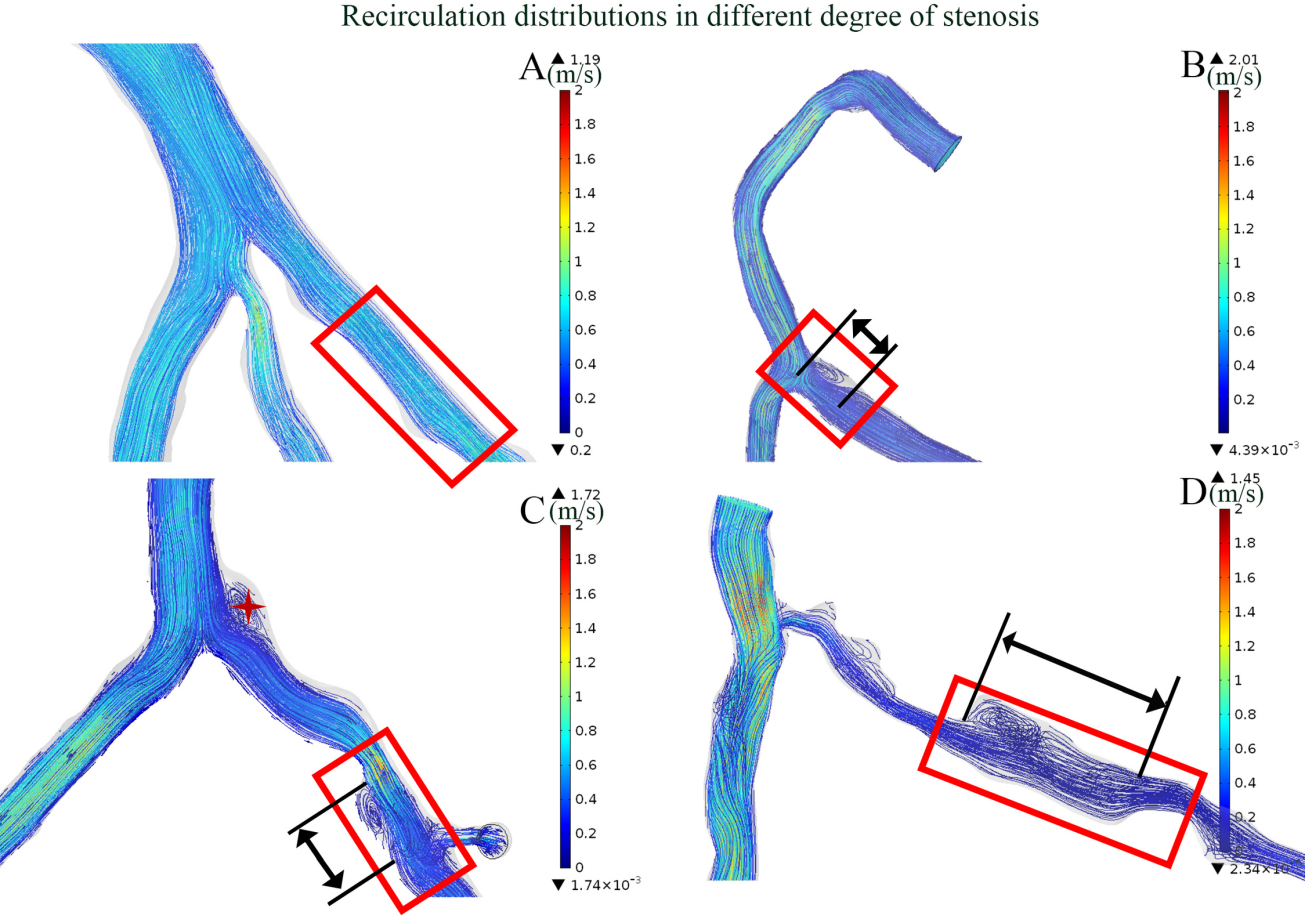
The effect of stenosis severity on the recirculation zone. Streamline of the flow distribution are illustrated (unit: m/s). The length the recirculation increase with the severity of the stenosis. Severity of the stenosis in A, B, C and D is 28.2%, 57%, 76.6% and 86.3% with the corresponding length of the recirculation zone is 0 mm, 5.02 mm, 6.48 mm to 16.77 mm, respectively. Recirculation zone is not only seen at the downstream of the stenosis, but also at the upstream bifurcation (labeled with red cross-star in C).

**Fig 7:**
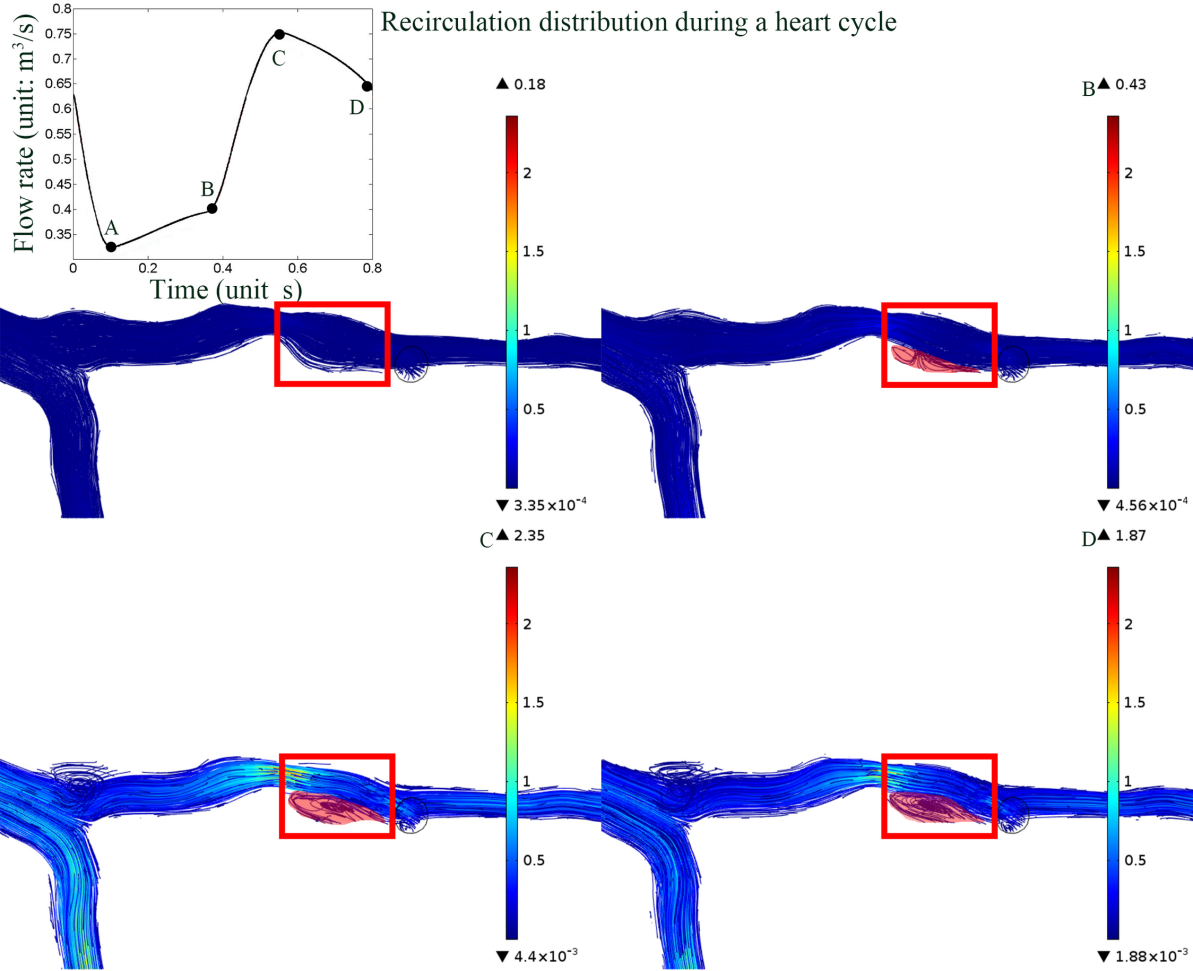
The streamline of the flow distribution color-coded with velocity magnitude. The pulsatile effect of the blood flow on the distribution of the recirculation. A, B, C and D illustrated 4 time points during one cardiac cycle. A: The recirculation disappeared at the low flow rate; B, C and D: The area of the recirculation zone varied along with the flow rate while the lengths of the recirculation zone were relatively consistence (5mm).

The length of the recirculation zone was measured as showed in Fig 6. The ends of the recirculation zone were labeled and then the value of the length was then measured from the geometry. The increasing severity of the stenosis contributes to the increasing length of recirculation in particular cases (as showed in Fig 6). The degrees of stenosis were 28.2%, 57%, 76.6% and 86.3% in cases A, B, C, and D, respectively. As the stenosis severity increased, the recirculation zone length increased from 0mm, 5.02mm, 6.48mm to 16.77mm (an increase of 454%, comparing Fig 6D with Fig 6B), respectively. The pulsatile effect of the blood flow to the distribution of the recirculation zone was analyzed as showed in Fig 7. The recirculation zone disappeared when the velocity was relatively low during the systolic period (Fig 7A). The area of the recirculation varied along the cardiac cycle and the largest was found at the peak flow rate (marked in red as showed in Fig 7). However, the length of the recirculation zone was consistence (5mm). On the other hand, those cases without recirculation at the peak flow period showed no response to the pulsatile flow.

**Fig 8:**
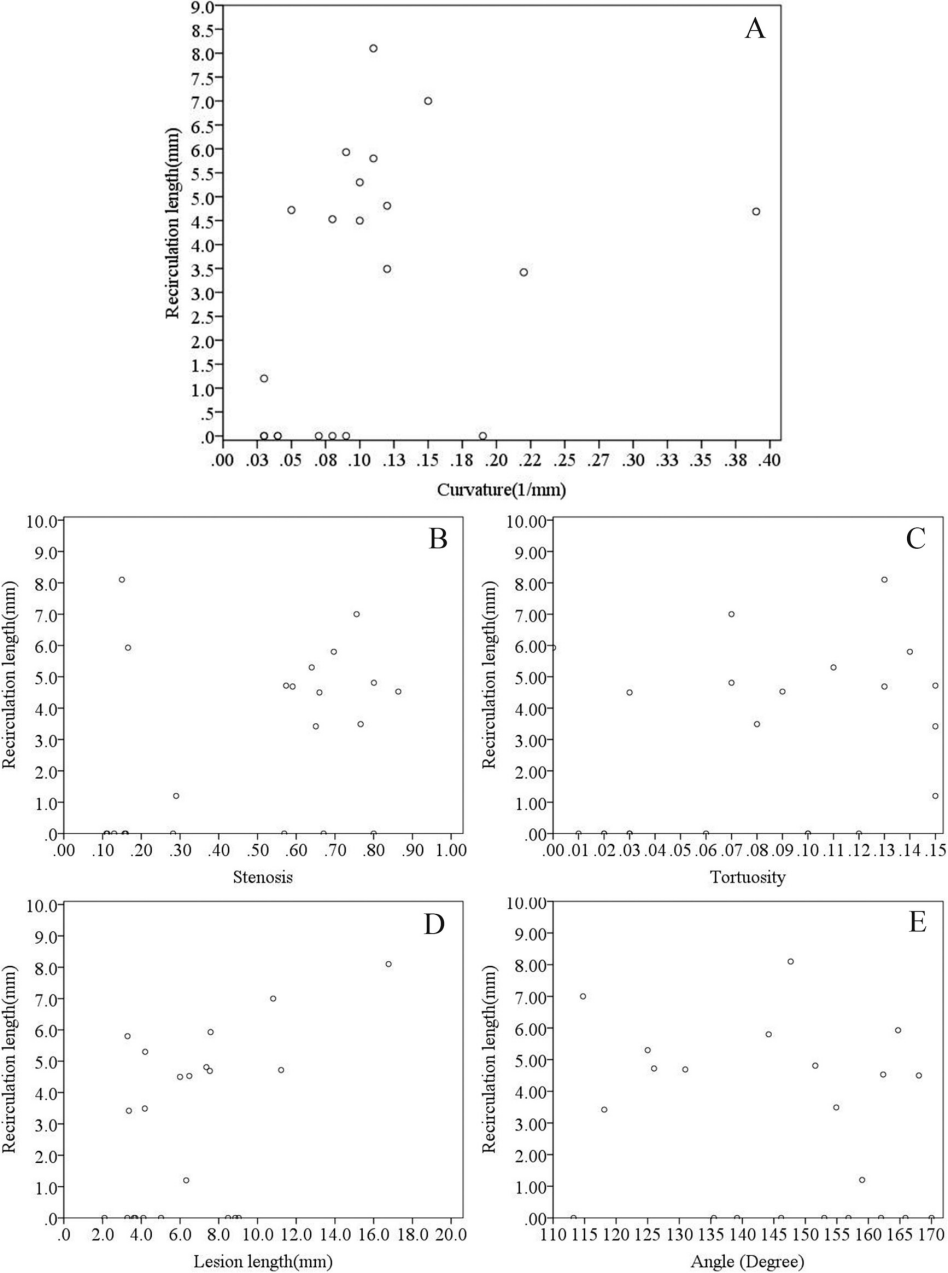
Relationship among stenosis severity, tortuosity, curvature, angle, lesion length with length of recirculation zone in the reconstructed patient-specific coronary arteries. A: effect of curvature on maximum wall shear stress; B: effect of stenosis severity (percent diameter stenosis) on maximum wall shear stress; C: effect of tortuosity on maximum wall shear stress; D: effect of lesion length on maximum wall shear stress; E: effect of angle of the lesion segment on maximum wall shear stress.

The relationship between the recirculation zone and geometric characteristics is illustrated in Fig 8. The length of the recirculation The recirculation length shows a close correlation with the curvature at the range of 0.03 to 0.15 (1/mm) (r = 0.505, P < 0.05). On the contrary, the severity of the stenosis (r = 0.316, P = 0.151, Fig 8B) and the length of lesion (r = 0.419, P = 0.052, Fig 8E) were weakly correlated with the recirculation length when neither the tortuosity (r = 0.262, P = 0.238, Fig 8C) nor the angle (r = -0.212, P = 0.345, Fig 8D) correlated with the recirculation zone length.

### Effect of bifurcation stenosis on the flow distribution and shear rate

**Fig 9:**
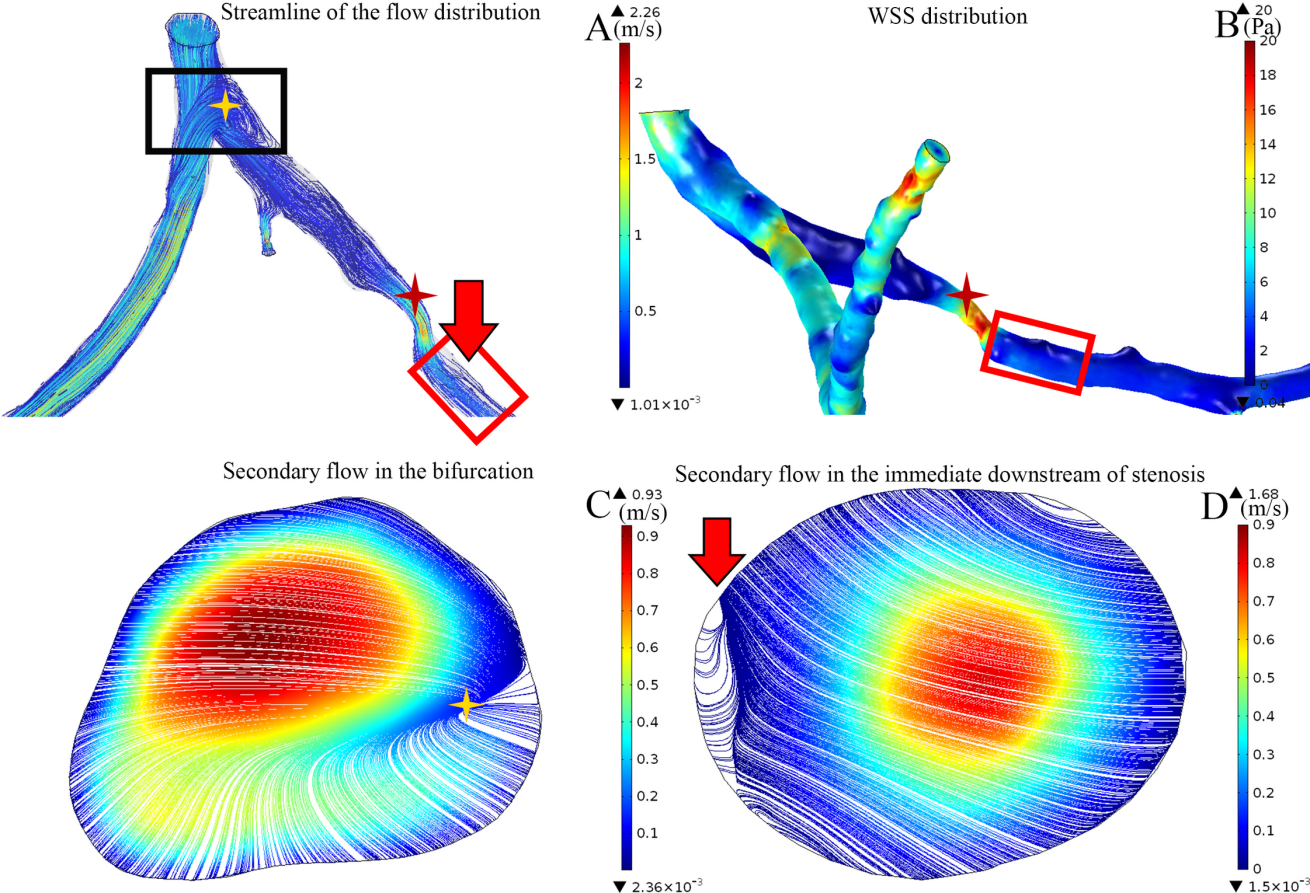
The distribution of the flow and the corresponding WSS distribution in the stenotic coronary artery. A: The streamline of the flow distribution color-coded with velocity magnitude showed that blood flow is separated at the downstream of the stenosis, leading to the occurrence of the recirculation zone (red arrow) (unit: m/s). Severe stenosis (>80%) resulted in the increasing of the resistance of the vascular bed, leading to the flow reversal at the upstream bifurcation (yellow cross-star). B: The vessel wall shear stress distribution showed that the maximum wall shear stress is found at the distal stenosis (red cross-star), disturbance of the flow is found in downstream, leading to the altered distribution of the wall shear stress (red frame) (unit: Pa). C and D: The streamline of the flow distribution color-coded with velocity magnitude in the cross-section area showed a more significant secondary flow pattern at the upstream (as in C labeled with yellow cross-star corresponding to the location showed in A) compare to that at the downstream of distal stenosis (as in D labeled with red arrow corresponding to the location showed in A) (unit: m/s).

The recirculation zone was found not only downstream of the stenosis but also at the bifurcation. The recirculation zones appeared at the bifurcations in 19 of the 22 cases in this study. As illustrated in Fig 9, a case was taken as an example. A mild flow reversal (as labeled with the red arrow in Fig 9A and 9D) was found at the untreated 57% degree of stenosis in the left anterior descending coronary artery (labeled with a red cross-star in Fig 9A). A high WSS was found at the stenosis, and a low WSS was found immediately downstream of the stenosis (Fig 9B in the area of the red frame). Moreover, there appeared a larger recirculation zone at the bifurcation upstream (Fig 9A in the area of the black frame). Although the secondary flow was insignificant downstream of the stenosis (Fig 9D labeled in red arrow), a larger recirculation zone was found at the left main coronary (LM) bifurcation. The streamline shows that the flow is separated from the LM into the efferent arteries, left anterior descending branch (LAD) and left circumflex branch (LCX), reversal flow from the LAD from the bifurcation and joint with the flow into the LCX where secondary flow is found, and the flow distribution is illustrated in Fig 9C (labeled with a yellow cross-star)

## Discussion

While the pressure distribution of in the stenotic coronary artery is mainly depend on the severity of the stenosis as the statistical analysis showed non-significant correlations between the geometric factors and the FFRCT in the stenosis in the present study. The geometric characteristics contribute to the hemodynamic redistribution in diseased human coronary arteries, involving a complex relationship between stenosis severity and curvature of the lesion segment. Stenosis severity is associated with maximum WSS while the curvature of the lesion segment is associated with the length of the recirculation zone. Although the data showed that the angle of the immediate downstream of the lesion, tortuosity, and lesion length all have a weak effect on the maximum WSS and length of the recirculation zone, it is important to consider the contribution of these geometric characteristics for a comprehensive analysis of the flow distribution and confirmation of the impact on the further development of the disease.

### Effect of the geometric characteristics on the WSS and recirculation

The severity of the stenosis is the main concern for diagnosing the significance of the coronary lesion [27,28]. However, other geometric characteristics are important in medical decision-making. The geometric characteristics varied between individuals. As showed in Fig 5A, the WSS increased along with the severity of the stenosis, and the dispersed distribution indicated that complex geometric factors co-contribute to the hemodynamic variations. Previous studies have shown that lesion length is highly accurate in evaluating functional ischemia in intermediate lesions [14]. Our result indicated that an increased lesion length contributes to the increased length of the recirculation zone (r = 0.401, P = 0.065). There are several cases presenting insignificant recirculation at the stenosis. The non-zero length of the recirculation zone showed a significant correlation to the lesion length of the corresponding cases (r = 0.608, P = 0.028). The curvature of the artery segment is another factor that concerns clinical treatment. As the curvature in the artery could cause helical flow pattern that response to the recirculation area [29] and the distribution of the WSS [30], the results of the present study showed that curvature demonstrates a positive correlation with both the maximum WSS and the length of the recirculation zone (Figs 5C and 8A). On the other hand, because the coronary arteries conform to the myocardium, there exist particular locations of curvature along the coronary arteries [31]. The stents and scaffolds implanted during invasive treatment of coronary stenosis via percutaneous coronary intervention (PCI) procedures consider the curvature because the implantation alters blood rheology, leading to early and late stent failure [32, 33].

### Effect of the bifurcation on the WSS and recirculation

**Fig 10:**
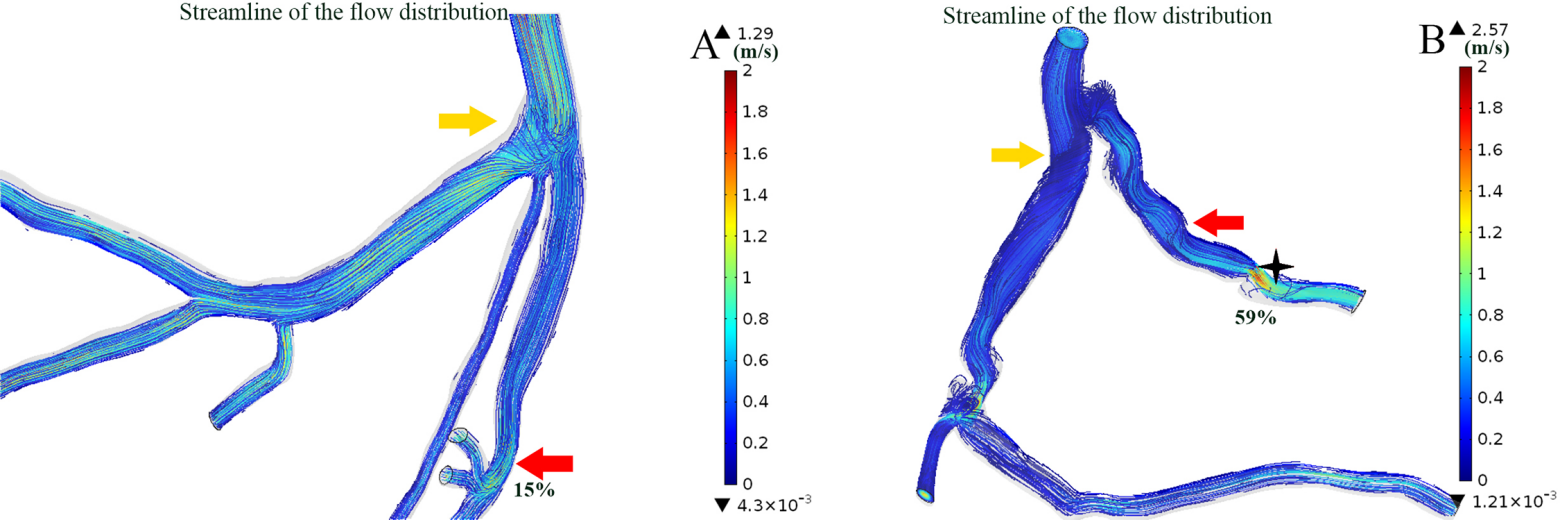
The streamlines of the flow distributions color-coded with velocity magnitude. Increasing of the resistance due to the distal stenosis leads to the flow reversal at the upstream bifurcation that blood flow is redistributed to the side branch as showed in A (unit: m/s). Examples of the disappearing of the recirculation zone at the downstream of the stenosis as showed in B (unit: m/s).

As noticed in the present study, the maximum WSS was significantly lower under the condition of the severe stenosis (>80%) in particular cases, as illustrated in Figs 5 and 9. The bifurcation at the LM coronary to LAD and LCX served as a divider to the blood flow. Severe stenosis at the LAD had increased the resistance of the vascular bed, resulting in side branch steal [34,35]. Flow reversal was also not seen in the stenosis in other cases, as shown in Fig 9 (labeled in red arrows), but it appeared at the bifurcation of the LM to LAD and LCX. Moreover, stenosis in the distal LAD (labeled with the black cross-star in Fig 10B) contributed to the increasing resistance, which prevented the occurrence of recirculation at the proximal stenosis [36]. Therefore, the distal condition of the vessel may cause hemodynamic alteration to the upstream stenotic region. Thus, this may be an explanation for the poor prognosis in particular bifurcation stenting [34]. There are other geometric indexes that show a correlation with the recirculation that is not included in the present study. A previous study showed that the increasing eccentric index results in an increased length of the recirculation zone [1].

### Limitations

There are certain limitations in the present study. The correlation factor between FFRCT and FFR was 0.987 with a P value < 0.01 except that there is an outlier as showed in Fig 1B. The FFRCT value of the outlier was 0.90 while the corresponding FFR value was 0.84. The diversion could indicate that it requires adjustment to the boundary condition method to meet the better agreement with the individual geometries. On the other hand, the assumptions of our model of CFD are laminar flow, Newtonian fluid, and a rigid wall. Because the coronary arteries are conforming on the heart wall, end-diastolic deformation of the vessel wall is not included. However, previous studies have suggested that the effect of these assumptions on the simulations is minor in stenotic vessels due to increasing in stiffness [37]. Although the sample size in the present study is relatively small, the results showed that stenosis and curvature significantly contribute to the WSS and recirculation, respectively.

## Conclusion

We performed hemodynamic analysis in 3-dimensional, patient-specific coronary arterial geometries with stenosis. The effect of the geometric factors on the flow distribution was investigated in the present study. The maximum WSS increased with the severity of stenosis. Moreover, the curvature of the lesion and lesion length co-contributed to the length of the recirculation zone. In contrast, the bifurcation of the coronary artery is also an important geometric factor such that stenosis at the distal vessel leads to flow redistribution to the side branches. These findings are relevant to the pathogenesis of arterial thrombosis and prognosis of the intervention treatment.

## Supporting Information

S1 File The CTA images of the patient 1. (RAR)

S2 File The CTA images of the patient 2. (RAR)

S3 File The CTA images of the patient 3. (RAR)

S4 File The CTA images of the patient 4. (RAR)

S5 File The CTA images of the patient 5. (RAR)

S6 File The CTA images of the patient 6. (RAR)

S7 File The CTA images of the patient 7. (RAR)

S8 File The CTA images of the patient 8. (RAR)

S9 File The CTA images of the patient 9. (RAR)

S10 File The CTA images of the patient 10. (RAR)

S11 File The CTA images of the patient 11. (RAR)

S12 File The CTA images of the patient 12. (RAR)

S13 File The CTA images of the patient 13. (RAR)

S14 File The CTA images of the patient 14. (RAR)

S15 File The CTA images of the patient 15. (RAR)

S16 File The CTA images of the patient 16. (RAR)

S17 File The CTA images of the patient 17. (RAR)

S18 File The CTA images of the patient 18. (RAR)

S19 File The CTA images of the patient 19. (RAR)

## Abbreviations

CAD: coronary artery disease
CTA: computed tomography angiography
ICA: invasive coronary angiography
FFR: fractional flow reserve
FFRCT: computed tomography-based FFR
CFD: computational fluid dynamics
LM: left main coronary artery
LAD: left anterior descending branch
LCX: left circumflex branch
